# Automated Segmentation of Mass Regions in DBT Images Using a Dilated DCNN Approach

**DOI:** 10.1155/2022/9082694

**Published:** 2022-02-02

**Authors:** Jianming Ye, Weiji Yang, Jianqing Wang, Xiaomei Xu, Liuyi Li, Chun Xie, Gang Chen, Xiangcai Wang, Xiaobo Lai

**Affiliations:** ^1^First Affiliated Hospital, Gannan Medical University, Ganzhou, China; ^2^School of Medical Technology and Information Engineering, Zhejiang Chinese Medical University, Hangzhou, China; ^3^Department of Medical Engineering, The 73th Group Army Hospital of P.L.A, Xiamen, China

## Abstract

To overcome the limitations of conventional breast screening methods based on digital mammography, a quasi-3D imaging technique, digital breast tomosynthesis (DBT) has been developed in the field of breast cancer screening in recent years. In this work, a computer-aided architecture for mass regions segmentation in DBT images using a dilated deep convolutional neural network (DCNN) is developed. First, to improve the low contrast of breast tumour candidate regions and depress the background tissue noise in the DBT image effectively, the constraint matrix is established after top-hat transformation and multiplied with the DBT image. Second, input image patches are generated, and the data augmentation technique is performed to create the training data set for training a dilated DCNN architecture. Then the mass regions in DBT images are preliminarily segmented; each pixel is divided into two different kinds of labels. Finally, the postprocessing procedure removes all false-positives regions with less than 50 voxels. The final segmentation results are obtained by smoothing the boundaries of the mass regions with a median filter. The testing accuracy (ACC), sensitivity (SEN), and the area under the receiver operating curve (AUC) are adopted as the evaluation metrics, and the ACC, SEN, as well as AUC are 86.3%, 85.6%, and 0.852 for segmenting the mass regions in DBT images on the entire data set, respectively. The experimental results indicate that our proposed approach achieves promising results compared with other classical CAD-based frameworks.

## 1. Introduction

Breast cancer is one of the leading causes of diseases in women worldwide, and it is also the most common cause of cancer deaths in women. Since the late 70s of the last century, the incidence of breast cancer worldwide has been increasing. According to the report “The Status and Trends of Cancer in China 2017” released by the National Cancer Center, the incidence of breast cancer ranks first among female malignant tumours [1]. Early diagnosis and treatment can effectively reduce the mortality of breast cancer patients and improve their quality of life [2]. In developed countries, organized and opportunistic screening programs have significantly reduced breast cancer mortality. Although two-dimensional mammography uses a new detector, it is well known that it still has its limitations because the normal structures and pathological structures may overlap each other when obtaining the transmission X-ray image [3].

Digital breast tomosynthesis (DBT) is a quasi three-dimensional imaging technology. An X-ray tube rotates in a limited arc, and a digital detector obtains a series of low-dose projection images to reconstruct the tomographic images [4]. DBT can evaluate the dense breast tissues in detail by describing the breast tissues in three dimensions to overcome the limitation that standard mammography only displays a two-dimensional image. In addition, the reconstructed DBT slice images can partially reduce the often called “anatomical” or “structure” noise caused by tissue superimposition in conventional mammography [5]. It is crucial to segment the breast mass regions in DBT slice images to provide accurate radiologists' qualification. Although DBT has higher sensitivity and specificity in breast mass regions detection, it has dramatically increased the amount of data in the image, resulting in manually annotating breast mass regions in DBT images. It is not only tedious but also time consuming for radiologists [6]. Hence, it is significant to develop a computer-aided segmentation framework for DBT mass regions to aid radiology clinicians to reduce the workload of manual annotation for the radiologists.

Automatic segmentation of breast mass regions is challenging because the breast mass regions have low contrast differences among their neighboring tissues [7]. Although it is difficult to achieve accurately segment breast mass regions, many studies focus on designing various automatic or semi-automatic learning-based approaches for breast mass regions segmentation in recent years [8–10]. Whereas automatic segmentation of breast masses from two-dimensional (2D) mammography has been widely investigated, little has been reported on segmenting breast mass regions automatically for DBT slice images. For each mass region, eight shape parameters and ten enhancement texture features were extracted and then an artificial neural network was used to build the diagnostic model; the average area under the receiver operating curve (AUC) reported by the system was 0.76 [11]. Breast tissues detection results were obtained using the multivariate statistical analysis of mass spectrometer data with a sensitivity of 90.9% and specificity of 98.8% [12]. If two-dimensional projected slice images and three-dimensional reconstructed volume can be combined for analysis, a computer-aided diagnosis (CAD) system of DBT can produce a lower false-positive rate [13]. In addition to research on breast segmentation by maximizing the radial gradient index in three dimensions of Reiser et al. [14], some publications only used a single representative two-dimensional slice [15, 16] to quantitatively evaluate the accuracy of mass regions segmentation in DBT images. Chan et al. [17] presented an approach on automatic detecting for breast mass regions. van Schie et al. [18] proposed an automated segmentation approach of breast mass regions, which used the mammography image data set to train the models. Kim et al. [19] concentrated on the influence of the saliency of the reconstructed slices on DBT mass regions' detection performance and then presented an automated detection system of breast mass regions based on the saliency of DBT reconstructed slices. Palma et al. [20] built a CAD framework based on the antagonistic reasoning and fuzzy theory, which can detect breast mass regions in reconstructed DBT images.

In recent years, deep learning has been successfully utilized in various medical image recognition tasks, such as tumour boundary detection, region segmentation, and pattern classification, because it does not adopt various handcrafted features in supervised manners [21–23]. The ResNet-50 model pretrained with transfer learning and class activation map technique were employed in breast cancer classification and localization, resulting in an AUC of 0.96 [24]. Sampaio et al. [25] proposed a computational methodology, where the quality of mammography image was improved initially by preprocessing, and the external region of breast was removed to reduce noise and highlight the internal structure of breast; next, the region was segmented and shape descriptors (such as eccentricity, circular disproportion and density) were extracted by using cellular neural networks, followed by an SVM classifier; they reported a sensitivity of 80% and AUC of 0.87. Wichakam and Vateekul [26] used support vector machines (SVMs) with ConvNets to detect mass on mammograms, where the reported accuracy was 98.44%, which was superior to the baseline (ConvNets) by 8%.

In this study, we combine DBT, a breast cancer screening method, with the latest machine learning and deep learning technologies. We focus on the accurate segmentation of the mass regions in DBT images using a deep convolutional neural network (DCNN) architecture, which is a fully convolutional network with dilated filters (dilated DCNN) instead of pooling filters. Besides, instead of training the model with the whole image, we implement a patch-based training approach. To produce an end-to-end segmentation output, we apply a fully convolutional network approach. Our system is used to every slice in a volume separately.

The structure of the paper is as follows: In Section 2, the data sets used and dilated DCNN architecture are introduced in detail, and then the training and testing methods of the dilated DCNN model for the mass regions segmentation in DBT images are introduced; Section 3 provides the details and results of the experiments performed in this study; Section 4 introduces the discussion, and the paper ends with Section 5, in which the conclusions and future work are presented.

## 2. Methodology

In this section, we will describe the data set used, the sampling procedure for generating input image patches, the architecture of the dilated DCNN, and the strategy for training the dilated DCNN, followed by the approach applied to segment the mass regions in DBT images and the evaluation metrics used.

**Figure 1:**
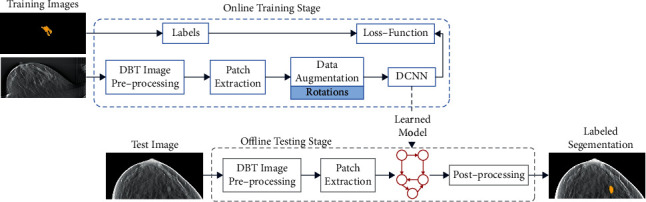
Overview of the proposed dilated DCNN approach.

A fully automatic framework for mass regions segmentation in DBT images is developed, an overview of the proposed architecture is shown in Figure 1. We apply a fully convolutional network approach to produce an end-to-end segmentation output. Our system is used to every slice in a volume separately.

### 2.1. DBT Image Data sets

The benchmarking clinical DBT image data used in this study are obtained from the Zhejiang Chinese Medical University Affiliated Guangxing Hospital (DBT_gx) and Zhejiang Provincial Hospital of Traditional Chinese Medicine (DBT_tcm) with Institutional Review Board (IRB) approval. Each DBT volume is produced by low-dose exposure, and the total shot dose should be within the range of a regular mammogram dose. Each patient's DBT data is acquired in medio-lateral oblique and cranio-caudal views (Siemens Mammomat Inspiration DBT system), using a total tomographic angular range of 60° with a 5° rotation increment and 12 projection views. Both sets of DBTs are reconstructed to 1 mm spacing slice with a resolution of 1200 × 901 pixels using the simultaneous algebraic reconstruction technique. We convert the images into TIFF stack/slices and used data in TIFF format to keep more details.

A total of 66 cases of breast cancer patients is included with a mean age of 53.65 years and an age range of 28–70 years. The entire DBT data set includes 146 views from 73 breasts with 97 masses, with size ranged from 4.7 to 37.8 mm (mean = 16.3 mm, median = 17.6 mm). Among them, 42 are benign lesions (absolutely healthy) and 55 were malignant lesions as determined by biopsy with subsequent histopathologic analysis. For each view (medio-lateral oblique or cranio-caudal), the number of slices ranged from 50 to 80 (mean = 69, median = 61). Two experienced radiologists manually annotate 97 masses and compared their annotations to reduce possible subjective errors. If there are any inconsistencies, the correct annotation of the mass regions in the image is determined by doctor consultation.

### 2.2. Slice Image Preprocessing

**Figure 2:**
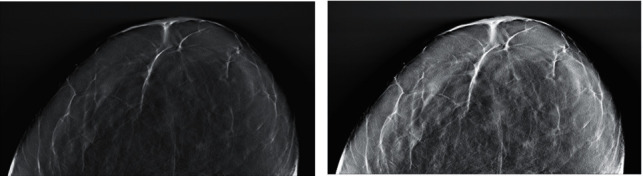
DBT image preprocessing results before and after preprocessing procedure. (a) DBT image before preprocessed. (b) DBT image after preprocessed.

For a typical DBT screening system, radiation exposure is a vital factor to avoid the risk of radiation-induced cancer. Hence, the low radiation dose is usually used to generate the DBT images. However, the total radiation dose of DBT is slightly higher than that of standard mammography. Generally, DBT image generally contains Poisson distribution noise. Considering the top-hat transformation can not only preserve more local details and highlight the hidden information but also suppress noise amplification. DBT images are preprocessed before the input patch extraction step, including using the top-hat transformation to enhance the contrast between the candidate breast mass location regions and background tissues. Furthermore, to improve the low contrast of breast tumour candidate regions and depress the background tissue noise in the DBT image effectively, a constraint matrix generated by an isotropic radial basis function centered on the candidate breast mass location region with a variance *δ*^2^ (*δ* is 5 mm) is established and multiplied with the DBT image.  Figure 2 shows the DBT image preprocessing results before and after preprocessing procedure, where Figures 2(a), 2(b) are the DBT image before and after preprocessed, respectively.

### 2.3. Input Patch Extraction

**Figure 3:**
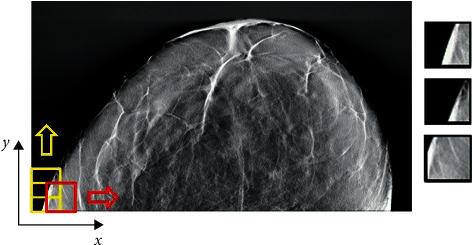
Sliding window method to scan the entire DBT image data and extract all possible input patches.

In our research work, compared with other DCNN-based detection problems, the DBT data sets available have a small number of image samples, so using the whole image directly is likely to result in overfitting. We split the entire DBT images into patches to address this issue, which increases data set dimension and complexity. As shown in Figure 3, we apply the sliding window method to scan the entire DBT image data and extract all possible input patches. All the patches are then classified according to the ground truth provided in the data sets. If the central pixel of an input patch is in the breast mass region, the input image patch is marked as positive (breast mass region candidate); Otherwise, it is designated as a negative (no mass region) label. Because the no mass regions can also provide valuable information for breast mass regions segmentation in DBT images, we extract image patches from breast mass regions and no breast mass region to augment the training image data. In other words, we use the image patches extracted from the no breast mass regions as the additional negative sample of dilated DCNN architecture training to help the proposed model distinguish the confounding regions and breast mass regions in the DBT images.

### 2.4. Dilated DCNN Architecture

**Figure 4:**
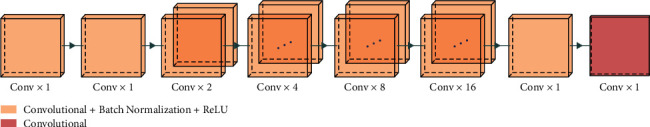
The proposed DCNN architecture.

This part will briefly introduce the architecture of the proposed dilated DCNN model and its application to our DBT mass regions segmentation framework. One of the problems of traditional typical CNN architectures using the max-pooling technique is that they will down-sample the image, resulting in segmented output with a resolution smaller than the input size. As shown in Figure 4 and Table 1, we use a new convolution network architecture, using dilated convolutions specially designed to get dense segmented output. The purpose of this model is to combine the multiscale context information systematically without losing the resolution, which is based on the dilated convolution to support the exponential expansion of the received field without losing the resolution or coverage.

Let *f*: Z^2^ be a discrete function. Let Ω_*k*_=[−*k*, *k*]^2^*Z*^2^, and let *r*: Ω_*k*_⟶*R* be a discrete filter of size (2*k*+1)^2^. The discrete convolution operator can be defined as

(1) $$\begin{array}{c} \left(f*r\right)\left(p\right)=\sum_{s+t=p}f\left(s\right)r\left(t\right). \end{array}$$

We now generalize this operator. Let *d* be a dilation factor and let *∗*_*d*_ be defined as

(2) $$\begin{array}{c} \left(f{*}_{d}r\right)\left(p\right)=\sum_{s+dt=p}f\left(s\right)r\left(t\right). \end{array}$$

We will refer *∗*_*d*_ as a dilated convolution or *d*-dilated convolution.

### 2.5. Patch-Based Training

The DBT image data sets used in our work exhibit serious class imbalance, i.e. the number of pixels in no breast mass regions is far more than that in breast mass regions. This brings a problem to the model's training because the pixels in the no breast mass regions influence the total loss function more than those in the breast mass regions. To settle this problem, we adopt an image patch-based training approach. In the training process, we balance the training image data by randomly resampling the same number of image patches of every class from all possible patches in each epoch. However, breast mass regions segmentation still has a similar problem of class imbalance. The number of positive samples is far less than the number of negative samples. Therefore, we adopt *f*_*α*_-measure as cost function, which is also known as the Dice coefficient. Compared with conventional loss (e.g., mean square error), the *f*_*α*_-measure enforces a better balance between performance on positive and negative regions, and thus is suitable for the task of mass regions in DBT images (having unbalanced samples in mass regions and nonmass regions).

Denote *T* and *S* as the ground truth heatmap and the predicted heatmap, respectively. Let *M* represent the number of elements (pixels) in *T* and *S*, and the *f*_*α*_-measure-based loss function is defined in formula (3)

(3) $$\begin{array}{c} f_{\alpha }\left(S,T\right)=\frac{\left(1+\alpha^{2}\right)\sum_{i=1}^{M}s_{i}t_{i}}{\sum_{i=1}^{M}s_{i}+\sum_{i=1}^{M}t_{i}}, \end{array}$$

where *t*_*i*_ is the *i*th element of the ground truth heatmap and *s*_*i*_ is the *i*-th element of the predicted heatmap. In this paper, we set *α*=1 based on the number of pixels in mass regions to the number of pixels in nonmass regions. If *α* is set to a higher value, the model loss mostly comes from the error of the mass regions, thus ignoring the error of the nonmass regions; otherwise, if setting to a smaller value, the model does not pay enough attention to the mass region because of the class imbalance, which reduces the segmentation accuracy.

### 2.6. DBT Image Data Augmentation

Extend the image data and generate more training data from the original image data to improve the performance of the proposed dilated DCNN model. Typical applications of the DCNN model for medical image analysis and computer vision tasks, rotations, and translations are often used to augment the data. In our work, the DBT image data consist entirely of 2D image patches. Hence, translation operation cannot augment the image data because it will cause a different image patch to have a possibly different class label. However, using rotation operations of the image patches might give some performance improvements. Therefore, we perform the rotation operation by using angles multiple of 90^o^.

### 2.7. Segmentation Postprocessing

In DBT images, some small clusters may be mistakenly classified as the breast mass regions. To deal with the problem, we impose a constraint by removing clusters in the segmentation output obtained by the proposed dilated DCNN-based system with less than 50 voxels in volumes.

### 2.8. Performance Analysis

To compare and analyze the performance with other classical CAD-based frameworks, the evaluation metrics used in this paper are (a) accuracy (ACC), sensitivity (SEN), and specificity (SPE) of the model; ACC refers to the ratio of the number of pixels correctly segmented to the number of total pixels in the image, SEN refers to the likelihood of a positive test among the subjects with the condition, and SPE refers to the probability of a negative test among the subjects without the condition. These three evaluation metrics are defined as follows:

(4) $$\begin{array}{c} \text{ACC}=\frac{\text{TP}+\text{TN}}{\text{TP}+\text{TN}+\text{FP}+\text{FN}}, \end{array}$$

(5) $$\begin{array}{c} \text{SEN}=\frac{\text{TP}}{\text{TP}+\text{FN}}, \end{array}$$

(6) $$\begin{array}{c} \text{SPE}=\frac{\text{TN}}{\text{TN}+\text{FP}}. \end{array}$$

TP, FP, TN, and FN denote true positive, false positive, true negative, and false negative, respectively, (b) free-response receiver operating characteristic curve (FROC), and (c) the area under the receiver operating curve (AUC). The FROC is used to evaluate the performance of the segmentation system on the DBT_gx and DBT_zcm data sets and is plotted between the fraction of correctly identified lesions as true-positive rate and the number of false-positive per volume (FPV) for all decision thresholds.

## 3. Results

### 3.1. Experimental Details

In this section, experiments with different training data sets are performed to evaluate the performance of the proposed dilated DCNN model. Note that in all cases, the original resolution of the processed DBT image is used. Patch-level data sets containing image patches of size 256 × 256 pixels are generated and used as the proposed dilated DCNN model inputs. In all experiments, a stride of 28 × 28 pixels is used to create the input image patches for training the dilated DCNN. The selection of the stride value is balanced according to the computational requirements and the number of training samples.

We apply the machine learning library Keras to implement the training and testing of the dilated DCNN model in Python 3.6. The training and testing experiments are performed on an NVIDIA Geforce Titan RTX 24G GPU with Intel Xeon Silver 4210 2.2 G GPU. The presented figures are generated using the plotting library matplotlib. To train the dilated DCNN, we used a batch size of 150 input image patches, 1000 batches per epoch, and 1000 epochs. We used an Adagrad optimizer [27] with a learning rate of 0.01.

### 3.2. Evaluation on Test Data sets

**Figure 5:**
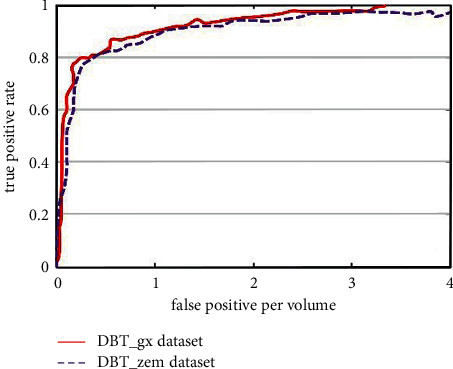
FROC curves for mass segmentation in DBT images on DBT_gx data set and DBT_zcm data set, respectively.

Given the limited data sets available in our study, mass regions segmentation in DBT images is performed using a ten-fold cross-validation strategy.  Figure 5 presents FROC curves measuring the proposed dilated DCNN model trained on the DBT_gx and DBT_zcm data sets, respectively. Our dilated DCNN-based model's performance is substantially higher when the framework is tested on the DBT_gx data set with TPR=0.971 ± 0.029 at 3.3 FPV, compared with that obtained on the DBT_zcm data set with TPR=0.937 ± 0.008 at 4.0 FPV.

**Figure 6:**
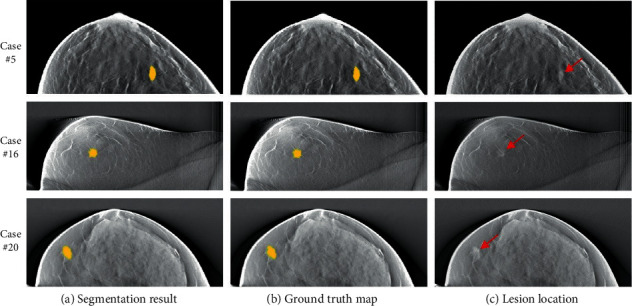
Illustration of mass segmentation examples in DBT images using proposed model compared with manual labeled. (a) Segmentation result. (b) Ground truth map. (c) Lesion location.

To further assess the mass regions segmentation performance in DBT images of our proposed method based on dilated DCNN, we evaluated the overlap between the proposed DBT mass labels and the ground truth maps.  Figure 6 illustrates mass regions segmentation in DBT images on a few testing images using our proposed model. The 1st column shows individual segmentation results with our proposed dilated DCNN model for case #5, case #16, case #20, the 2nd column shows the corresponding ground truth maps, and a red arrow is used to indicate the lesion without obscuring the lesion in the 3rd column, respectively. The examples shown in Figure 5 indicate that the mass regions segmentation outputs predicted by our proposed dilated DCNN model are in high agreement with the manual annotations.

## 4. Discussion

In this study, a dilated DCNN architecture is specifically designed for the dense prediction of mass regions in DBT images, which systematically aggregates multiscale contextual information without losing resolution. Our proposed model adopts the *f*_*α*_-measure as the cost function to suppress the influence of class imbalance. To generalize the applicability of the proposed dilated DCNN-based framework, we combine the two DBT image data sets into a larger data set, which is called the entire data set. In the experiments, we compare the performance of various typical automatic detection methods of breast mass regions in DBT images in terms of classifier used, DBT image data set size, SEN, ACC, and AUC. As can be seen in Table 2, our model based on dilated DCNN has achieved competitive results than some of them. Among these classic models, we will discuss the research work of Kim et al. [28], Fotin et al. [29], and Samara et al. [30] in detail. They applied deep learning to the detection and segmentation of breast mass regions in DBT images. Their research works evaluated the automated segmentation CAD frameworks for breast masses in DBT images using the hand-crafted feature- and DCNN-based models. The DCNN model proposed by Kim et al. [28] extracted low-level features from the regions of interest (ROIs) and corresponding ROIs, respectively, through the convolutional layers separately, which can recognize the latent bilateral feature representations of breast masses in the reconstructed DBT volumes. To represent the high-level bilateral features of breast masses in DBT images, they combined the low-level features into the fully connected layer. It was reported that the AUC of the latent bilateral feature representation model was 0.847. Fotin et al. [29] developed a DBT mass detection CAD framework using DCNN model. They trained the DCNN with the generated candidate ROIs, which included 1864 mammography breast lesions and 339 breast lesions from DBT images data. According to the report, 86.40% of ACC and 89% of SEN were obtained in their model. Samala et al. [30] proposed a DCNN framework composed of four convolutional layers and three fully connected layers. First, the DCNN model was trained on a large-scale two-dimensional mammography data set. The weights of the first three convolutional layers were frozen, and the rest weights continued to be trained and updated. Through the calculation results of the DCNN model, it can be seen that the AUC value was more than 0.8, and the SEN value was over 80%. As for the method proposed in our work, 87.1% ACC, 86.9% SEN, 88.2% SPE, and 0.859 AUC for the testing data set with 89 DBT volumes are obtained.

In other models not based on DCNN network, we select the studies of Chan et al. [17], van Schie et al. [16], Palma et al. [20], and Reiser et al. [31] for comparative analysis. Chan et al. [17] introduced three methods based on two-dimensional and three-dimensional, as well as the combination of two-dimensional and three-dimensional methods. For the data set contained 100 DBT images from 69 patients with malignant patient cases, they obtained 80% SEN and 1.23 FPs/volume using the hybrid method. van Schie et al. [16] proposed a two-stage method, the first step was to locate ROIs in 2D slice images, and the second step was to locate 3D ROIs on DBT volumes combined with the extracted regions in 2D slice images. The results obtained from the DBT image data of 49 patients with one or more malignant tumours in 192 cases showed that 80% of the Sen was 3 FPs/volume. Palma et al. [20] developed a dual-channel DBT masses detection CAD framework in which each channel classified a type of DBT lesions. They combined discoveries and disjunction fusion methods from the channels. Their results showed that 90% SEN of the 101 DBT volumes contained 53 lesions. Reiser et al. [31] introduced a method of detecting DBT breast mass in two-dimensional projection views, and then reported 90% SEN for 36 DBT volumes using the visual angle range found in combination with detections.

**Figure 7:**
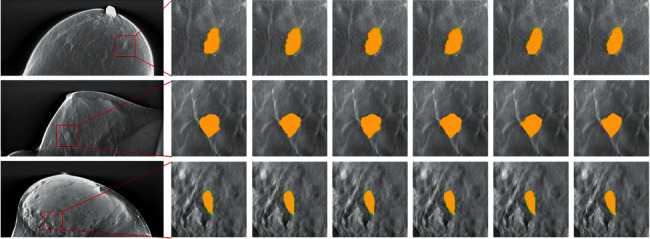
Examples of mass regions in DBT images segmented by our dilated DCNN framework and other typical CAD systems.

Figure 7 shows examples of automatic segmentation of breast mass regions in DBT images by our dilated DCNN architecture and other typical CAD architectures, which is obtained by overlaying the breast mass regions in DBT images segmented by our proposed architecture and other typical CAD architectures to the original image, respectively. We carefully studied these methods presented by Kim et al. [28], Fontin et al. [29], Samala et al. [30], and Reiser et al. [31], then we configurated the frameworks and developed these models in Python 3.6 with the machine learning library Keras and applied the models to our own data set in our study. Otherwise, it is unfair to make a comparison of the performance between our dilated DCNN model with other CAD models in automatic segmentation of breast masses in DBT images because other CAD models are trained and tested on different private data sets that are not available in public. Although the automatic segmentation model proposed in this paper cannot achieve the best overall DBT mass regions segmentation performance, our dilated DCNN framework achieves 86.3% ACC and 85.6% SEN with AUC of 0.852. The experimental results of this paper also indicate that our framework can get sound segmentation outputs on DBT image data set, and the dilated DCNN model is trained on the two-dimensional slice images of DBT volumes, not on the two-dimensional mammography data set. Although the proposed DCNN-based CAD framework has achieved promising results in automated segmentation of breast mass regions in DBT images, it can be further improved when there are more DBT image data. The main limitation of this study is the lack of sufficient DBT image data. To achieve satisfactory overall segmentation performance, our proposed automated segmentation framework for breast mass regions in DBT images needs more diverse data and structural distortion samples. Our proposed dilated DCNN-based approach can be applied to detect all early signs of breast tumour in DBT images, which is vital to decrease the review time for radiologists while maintaining or decreasing false positives.

## 5. Conclusions

This article presented a novel dilated DCNN-based architecture for mass regions segmentation in DBT images, performed experiments on an in-house collected DBT image data set, and obtained promising results. The constraint matrix is generated by using isotropic radial basis function and multiply with the DBT image to effectively improve the low contrast of candidate breast tumor regions and suppress the noise of background tissue regions. Our dilated DCNN architecture is specifically designed for dense prediction, systematically aggregating multiscale contextual information without losing resolution. Moreover, the proposed model adopts the *f*_*α*_-measure as the cost function, further effectively suppresses the influence of class imbalance, and can improve the generalization ability of the segmentation. The average ACC, SEN, AUC obtained on the entire data set are promising and they are 86.3%, 85.6%, and 0.852, respectively. This study demonstrates that the presented dilated DCNN network has the potential to segment the mass regions in DBT Images accurately.

## Figures and Tables

**Table 1:** The proposed dilated DCNN configuration and parameters.

Layer	Type	Configuration	Dilation	Number of parameters
1	Convolutional	3 × 3 × 1 × 32	1	2256
	Batch normalization			
	ReLU			

2	Convolutional	3 × 3 × 32 × 32	1	18464
	Batch normalization			
	ReLU			

3	Convolutional	3 × 3 × 32 × 32	2	36928
	Batch normalization			
	ReLU			

4	Convolutional	3 × 3 × 32 × 32	4	73856
	Batch normalization			
	ReLU			

5	Convolutional	3 × 3 × 32 × 32	8	147712
	Batch normalization			
	ReLU			

6	Convolutional	3 × 3 × 32 × 32	16	295424
	Batch normalization			
	ReLU			

7	Convolutional	3 × 3 × 32 × 32	1	295424
	Batch normalization			
	ReLU			

8	Convolutional	1 × 1 × 32 × 2	1	16384

**Table 2:** Comparisons of typical studies in mass regions detection of the DBT images.

Method	Classifier	DBT image data set size	Sensitivity	Accuracy	AUC
Chan et al. [17]	LDA	100	80%	/	/
van Schie et al. [18]	NN	752	80%	/	/
Palma et al. [20]	SVM	101	90%	/	/
Kim et al. [28]	SVM	160	/	/	0.847
Fotin et al. [29]	DCNN	344	89%	86.4%	/
Samala et al. [30]	DCNN	324	80%	/	0.80
Reiser et al. [31]	LDA	36	90%	/	/
Proposed	Dilated DCNN	97	85.6%	86.3%	0.852

## Data Availability

The raw/processed data required to reproduce these findings cannot be shared at this time as the data also forms part of an ongoing study.
